# Food Security, Dietary Intake, and Foodways of Urban Low-Income Older South African Women: An Exploratory Study

**DOI:** 10.3390/ijerph18083973

**Published:** 2021-04-09

**Authors:** Feyisayo A Odunitan-Wayas, Mieke Faber, Amy E Mendham, Julia H Goedecke, Lisa K Micklesfield, Naomi E Brooks, Dirk L Christensen, Iain J Gallagher, Kathryn H Myburgh, Angus M Hunter, Estelle V Lambert

**Affiliations:** 1 Research Centre for Health through Physical Activity, Lifestyle and Sport, Division of Exercise Science and Sports Medicine, Department of Human Biology, Faculty of Health Sciences, University of Cape Town, Cape Town 7700, South Africa; AE.Mendham@uct.ac.za (A.E.M.); Julia.Goedecke@mrc.ac.za (J.H.G.); Lisa.Micklesfield@wits.ac.za (L.K.M.); vicki.lambert@uct.ac.za (E.V.L.); 2 Non-Communicable Diseases Research Unit, South African Medical Research Council, Tygerberg 7505, South Africa; Mieke.Faber@mrc.ac.za; 3 SAMRC/Wits Developmental Pathways for Health Research Unit, Department of Paediatrics, School of Clinical Medicine, University of the Witwatersrand, Johannesburg 2000, South Africa; 4 Faculty of Health Sciences and Sport, University of Stirling, Stirling FK9 4LA, UK; n.e.brooks@stir.ac.uk (N.E.B.); i.j.gallagher@stir.ac.uk (I.J.G.); a.m.hunter1@stir.ac.uk (A.M.H.); 5 Section of Global Health, Department of Public Health, University of Copenhagen, 1014 Copenhagen, Denmark; dirklc@sund.ku.dk; 6 Department of Physiological Sciences, Stellenbosch University, Stellenbosch 7600, South Africa; khm@sun.ac.za

**Keywords:** older women, food-related decisions, coping strategies, well-being, nutrition

## Abstract

This cross-sectional study explored the differences in sociodemographics, dietary intake, and household foodways (cultural, socioeconomic practices that affect food purchase, consumption, and preferences) of food secure and food insecure older women living in a low-income urban setting in South Africa. Women (*n* = 122) aged 60–85 years old were recruited, a sociodemographic questionnaire was completed, and food security categories were determined. The categories were dichotomised into food secure (food secure and mild food insecurity) and food insecure (moderate and severe). A one-week quantified food frequency questionnaire was administered. Height and weight were measured to calculate body mass index (BMI, kg/m^2^). Most participants (>90%) were overweight/obese, unmarried/widowed, and breadwinners with a low monthly household income. Food insecure participants (36.9%) more frequently borrowed money for food (57.8% vs. 39.0%, *p* = 0.04), ate less so that their children could have more to eat (64.4%. vs. 27.3%, *p* = 0.001), and had higher housing density (1.2 vs. 1.0, *p* = 0.03), compared to their food-secure counterparts. Overall, <30% of participants met the WHO (Geneva, Switzerland) recommended daily servings of healthy foods (fruits, vegetables, and dairy products), but >60% perceived that they consumed an adequate amount of healthy foods. The overall low-quality diet of our cohort was associated with poor nutritional perceptions and choices, coupled with financial constraints.

## 1. Introduction

Food security and adequate nutrition are of great importance for the well-being of older adults, helping to reduce age-related vulnerability to disease, mental deterioration, and impaired immune function [1,2]. Studies have shown that the increasing prevalence of non-communicable diseases (NCDs) and obesity, especially in older adults, is linked to food insecurity, foodways, and unhealthy lifestyle behaviours [3,4]. Food security exists when ‘all people, at all times, have physical, social and economic access to sufficient, safe and nutritious food that meets their dietary needs and food preferences for an active and healthy life’ [5]. Foodways refer to the cultural, social, and economic practices of households that affect food consumption, food purchasing patterns, food choice, and preferences [6]. Food insecurity in older adults has been linked to financial vulnerability, low socioeconomic and educational status, overweight and obesity, being a female, living alone or with their children, reduced frequency of social contact, and lower intake of food energy and nutrients [2,4,7,8,9]. 

Neighbourhood food environments and household foodways, such as having adequate local food retail outlets, being able to shop independently for food, and being able to prepare food at home, can help to reduce food insecurity risk, especially for older adults [10]. Accordingly, food-insecure older adults often consume low-cost, highly processed, and energy-dense unhealthy foods, which may contribute to the high prevalence of obesity in these individuals [11,12]. Early studies in South Africa have shown that food-insecure households lack adequate storage facilities at home for bulk buying, and are more likely to use informal retail food outlets as they offer physical proximity and convenient types of foods [13]. However, more recently, purchasing food from major retail supermarkets has become increasingly common in South Africa, with higher socioeconomic status and food security associated with greater expenditure on unhealthy food options in contrast to healthier food purchases [10,14,15]. 

To our knowledge, no study has focused on factors associated with dietary patterns and food security, and how this is influenced by the food environment and household foodways in older women from a low-income setting. In South Africa, a large proportion of older adults, especially women, have the double burden of supporting and caring for their grandchildren and adult children, due to the high prevalence of HIV/AIDS, high unemployment, labour migration, and children born outside of marriage [16,17]. This may compromise their own dietary needs, food security, and foodways. Examining foodways, dietary intakes, and food security status of low-income older South African women may add to our understanding of factors influencing food choice behaviours that may impact their health. Accordingly, the objective of this cross-sectional study was to describe food security, dietary intake, and foodways of low-income older South African women, and to examine the differences in foodways and dietary intake between food secure and food insecure low-income older South African women.

## 2. Materials and Methods

### 2.1. Participants and Setting

This study was conducted in a low-income urban setting in Cape Town, South Africa from April 2018 to December 2018. A convenience sample of older women (*n* = 122) aged 60–85 years old, who were able to understand verbal and written information about the study, were living independently (living in their own household or living with family), and were ambulatory were recruited. We primarily recruited participants through senior adult community groups/clubs in Khayelitsha and Langa, which are low-income, predominantly black, urban areas in Cape Town. Khayelitsha has the highest poverty rate and is the largest and fastest-growing township in Cape Town, with a population of approximately one million people [18]. Langa is the oldest surviving township in Cape Town and is densely populated with a population of 50,000, according to the 2011 South Africa census population. These townships comprise both formal and informal housing [19]. 

### 2.2. Measures 

#### 2.2.1. Questionnaires

Questionnaires were interviewer-administered by clinical research workers in either one or a combination of English and Isi-Xhosa (two of the three major languages in Cape Town).

Sociodemographic questionnaire: Questions included information on house type (brick house or informal shack) and house ownership, the highest level of education completed, marital status, household density (ratio of inhabited rooms to the number of people living in the house), household assets such as refrigerator, stove, microwave, access to basic amenities (i.e., running water, flush toilet system, electricity), monthly household income (total amount of money earned monthly by members of the household), categorised as <ZAR 2500/USD 156 and >ZAR 2500/USD 156), number of children and adults (≥18 years old) in the household, and number of people (children and adults) in the household financially supported by household income.

Household food insecurity access scale (HFIAS): This nine-question validated instrument has been used extensively in South Africa [20,21]. These questions reflect concerns about access to food (Question 1), insufficient food quality (Questions 2–4), and insufficient food intake and its physical consequences (Questions 5–9) within the previous 30 days. Each question that elicits a ‘yes’ response is followed by a frequency-of-occurrence question with three options: ‘rarely’, ‘sometimes’, and ‘often’. Responses were scored (‘No’ = 0, ‘rarely’ = 1, ‘sometimes’ = 2, ‘often’ = 3), and the scores were summed. A higher score represents greater food insecurity. The continuous scores were divided into four categories, representing food-secure (0–1), mildly (2–8), moderately (9–16) and severely food-insecure (17–27) households, according to the categorisation scheme in the HFIAS Indicator Guide [20]. To create a binary variable, food secure and mildly food insecure categories were combined and classified as ‘food secure’, while moderately to severely food insecure categories were combined as ‘food insecure’. In addition, response to each HFIAS question (yes or no) was also assessed in percentages [20]. 

Food acquisition questionnaire: The food acquisition questionnaire (Supplementary Materials, Table S1) was adapted from the Slow, Stop, or Stem the Tide of Obesity in the People of South Africa (STOP–SA) questionnaire and the first South African National Health and Nutrition Examination Survey (SANHANES-1). It consisted of questions on the following:

(a) The major source of household income and other sources of household income;
(b) Household shopping and foodways: This includes questions about whether the participant is the person in the household who is responsible for shopping, food preparation, food budget, food type to purchase, frequency of shopping at different food outlets or places where they eat outside the home (i.e., supermarkets, spazas, street food vendors, fast food, social/faith-based clubs), food types purchased/consumed and estimated expenditure in these various food outlets, the major reason for choice of food outlets and mode of transportation to these places.

Supermarkets are recognised retail store chains in South Africa that offer a broad selection of foods and household products.

Spaza shops are small retail stores, often in a residential area, that carry a limited selection of items such as staples, junk food, and drugstore items, and which is open long hours for the convenience of shoppers. 

Street vendors are people who offer goods or services for sale to the public without having a permanently built structure but with a temporary static structure or mobile stall.

Fast food outlets offer foods or meals that are prepared or ready for immediate consumption either at the place of purchase or elsewhere. They are also known as takeaway outlets.

Social/faith-based clubs are informal/formal community organisations where members with common interest(s) go to meet.

(c) Coping strategies for food: This section asks whether participants borrow money for food, from whom they borrow money for food, and if they eat less than they should so that others in the family, especially the children, will have enough to eat;
(d) Perceptions on the consumption of healthy diet: This section asks about whether the participants think they have a healthy diet, and if when they compare their diet to a healthy diet, they perceive that they consume too much, too little, or about the right amount of food types such as fruits, vegetables, and dairy products.

Food frequency questionnaire: A quantified seven-day food frequency questionnaire (QFFQ), recording the frequency of foods consumed during the previous week, was administered by a trained research assistant. The food flashcards (high-quality photographs) were used to assist with providing a better description of the food items. A standardised ‘dietary kit’ that included examples of food containers, plastic food models, household utensils, and three-dimensional sponge models were used to help the participants describe the amount of food consumed [22]. Amounts reported in household measures or volume were converted to grams using the South Africa Medical Research Council (SAMRC) Food Quantities Manual for South Africa [23]. Food intake was converted to energy and nutrients using the South African food composition database [24]. Foods consumed were categorised into 12 food groups, as indicated in Supplementary Materials, Table S2, based on a recent South African study [25]. For each food group, energy contribution was calculated and expressed as a percentage of total energy intake (%TE). Additionally, the number of servings consumed per food group was calculated using standard serving sizes, using the South African food-based dietary guidelines as a guide [26]. Participants reporting an energy intake of <4000 kJ per day were excluded [27,28].

#### 2.2.2. Anthropometric Measurements

Height and weight were taken with participants wearing only lightweight clothing and without shoes. Height was measured (3PHTROD-WM, Detecto, MI, USA) and recorded to the nearest 0.1 cm. Weight was measured to the nearest 0.1 kg on a calibrated, electronic scale (BW-150, NAGATA, Tainan, Taiwan). The body mass index (BMI) was calculated as kilograms divided by meters squared and categorised as either underweight (<18.5 kg/m^2^), normal weight (18.5–24.9 kg/m^2^), overweight (25–29.9 kg/m^2^), or obese (≥30 kg/m^2^) [29]. 

### 2.3. Data Analysis

Data were analysed using IBM SPSS for Windows, version 26, Armonk, New York: IBM Corporation. Categorical data were presented as frequencies (percentages) and differences tested by chi-squared test or Fisher’s exact test. Normal distribution of continuous variables was tested using Shapiro–Wilk test. Normally distributed variables were presented as mean and standard deviation (SD) and compared between the food secure and food insecure groups using an independent *t*-test. Non-normally distributed variables were presented as median and interquartile range (IQR) and compared between groups with Mann–Whitney U test. All differences were considered significant at *p* ≤ 0.05. 

## 3. Results

In this sample of older (median age 67 years old) South African women, 63.1% were classified as food secure (15.6% were food secure, and 47.5% were mildly food insecure) and 36.9% food insecure (26.2% were moderately food insecure, and 10.7% were severely food insecure). The assessment of each of the HFIAS questions separately showed that more than half of the participants ‘worry that their household would not have enough food’ (71.7%), ‘eat just a limited variety of foods due to a lack of resources’ (59.3%) and ‘eat smaller amount than required due to insufficient amount of food’ (51.2%) (Supplementary Materials, Table S3). The demographics and household characteristics of the total sample and the food-secure and insecure groups are presented in Table 1. The majority of the women were either single, widowed, or divorced and heads of their households (92.6%). Most of the participants did not complete high school education (93.4%) and were overweight or obese (91%) with a median BMI of 33.3 kg/m^2.^ Approximately 75% had a monthly household income of <ZAR 2500/USD 156. The majority (>80%) of the cohort owned their residential house and lived in brick houses. Almost all (>94%), had access to a flush toilet system, electricity, and running water in their houses, and <15% owned a car. 

The majority of participants (>75%) had a refrigerator and stove. These factors did not differ between food secure and food insecure women however, housing density was higher in the food insecure households, who were also less likely to have a microwave than those who were food secure (73.3 vs. 99.3%).

Household and food decision characteristics of food secure and insecure women are presented in Table 2.

The median household size was five people, with at least one child and three adults in each household being financially supported with the household income, the primary source of which was from the government social grant pension of the participant (73.6%). Almost three-quarters (73%) of the participants care for their grandchildren daily and were responsible for deciding what food to purchase (77.9%), and how much to spend on food (79.5%). More than half were responsible for grocery shopping (52.5%) and food preparation (59.0%). These factors did not differ between the food secure and insecure groups. Compared to the food-secure group, a greater proportion of food insecure women borrowed money for food which was mostly from friends or neighbours (60%), ate less, so others, especially children in the household, could have more to eat (64.4% vs. 27.3%) and attended social/faith-based groups for socialisation which often involved having meals provided (82.2% vs. 63.2%). All the participants shopped at supermarkets, 88.4% shopped at spaza shops, and 73.6% at street food vendors, while 54.1% bought food from fast food outlets. These factors did not differ by food security status. The food acquisition characteristics are presented in Table 3. As there were no differences in these characteristics when categorised by food security status, they are presented for the total cohort.

All the participants shopped at supermarkets mostly due to their perceived low price, with more than half (59.5%) going at least twice a month. Grains/cereals and legumes, frozen meat/chicken, and fresh and frozen vegetables were the most frequently purchased food items at supermarkets. Nearly two-thirds (63.6%) of the participants bought food from spaza shops at least once a week, with 88.5% reporting that this was due to easy access (convenience). Food items frequently purchased at spaza shops included bread, dairy products, and condiments. Foods bought from street vendors were mostly fruits and vegetables, and women reported shopping at street vendors primarily due to the quality (47.9%) of the food items. Other factors such as convenience, low price, variety were <25%. Just over half of the participants bought food at fast food outlets, typically once a month. Almost half of the participants went to social/faith-based clubs at least once a week to socialise and have meals with other people. Walking was the most common mode of transportation for the participants to attend the social/faith-based club and food-specific outlets, except supermarkets, where 52.2% used public transport. 

The contribution of macronutrients and specific food groups to total energy (%TE) intake is presented in Table 4. 

The food-insecure participants consumed a significantly higher %TE from carbohydrates and lower %TE from fat than their food-secure counterparts. For both groups, total energy intake fell outside the acceptable macronutrient distribution range (AMDR). The AMDR is the range of intake for a specific energy source that is associated with reduced risk of chronic disease while providing intakes of essential nutrients [30]. Food group analysis showed that food-insecure participants had a higher %TE for legumes and a lower %TE from fats and oil. The proportion of the participants who consumed the different food groups and the median number of servings per day consumed for eight of the 12 food groups are shown in Table 5. These eight food groups presented are the healthier options of the food groups. Each of the food groups was consumed by more than 90% of the participants, except for legumes (73%) and nuts (39%), and this did not differ between the groups. Only a small proportion of the participants met the recommended number of servings for fruit (26.2%), vegetables (13.9%), and dairy products (5.5%). However, overall, 62.8% considered their diet to be healthy, while 17.4% ‘did not know’. The majority of the women, regardless of their food security status, considered their fruit (67.8%) vegetable (86.8%), and dairy (62.5%) intake to be adequate or more than adequate (Supplementary Materials, Table S4).

## 4. Discussion

In this convenience sample of older South African women who reside in a low-income, urban community, 36.9% of women were from households considered to be moderately or severely food insecure. Housing density was the only measure of socioeconomic status that differentiated the food-secure and -insecure groups, with access to basic amenities and the type of residential house that they lived in being similar between the groups. In the majority of cases, with no differences between the groups, the women in our study decided what food to purchase for the household, were the main household shopper, and were responsible for the household food budget and food preparation, while more women in the food-insecure group reported borrowing money for food and eating less so that the children in the household had more to eat. We also showed that different food items were frequently purchased from the various food outlets, and the reasons for this differed between the outlets. Despite most women reporting that they eat a healthy diet of fruit, vegetables, and dairy products, less than 30% met the recommended daily servings.

Food insecurity has been positively linked with poor dietary intake in low-income households [31,32]. Despite the food-secure group consuming more of their energy from fat, both groups’ %TE from fat was within the recommended AMDR, albeit in the lower range. The higher %TE from carbohydrate for the food-insecure group is possibly due to the combination of the significantly higher intake of legumes (high in carbohydrates and protein, and low in fat) [33] and slightly higher, although not significantly, intake of starchy grains, cooked porridge and sugar, and sugary foods. Notwithstanding, both groups had a high carbohydrate intake since more than half of their energy intake came from the consumption of starchy grains, cooked porridge and, sugar and sugary foods (low-cost, high energy-dense foods). Consequently, the calorie intake in both groups was slightly higher than the AMDR. Although the food types purchased and consumed is diverse in our study, the daily consumption of fruits, vegetables and dairy products was low (<30% met the recommendations), less than half (39.1%) consumed nuts and seeds, and less than 75% consumed legumes possibly due to difficulty in their digestion and the high cost of nut [34,35]. These findings are comparable to previous findings in older, low-income, previously disadvantaged South Africans, shortly after the end of apartheid [36,37]. Apparently, the trend of low intake of fat, fruits, and vegetables and a high intake of carbohydrates of this group remains largely unchanged notwithstanding food security status. This combination of low fruit and vegetable intake, combined with a high intake of processed carbohydrates, may be two factors contributing to the overweight and obesity prevalence of 91% in this sample, which did not differ by food security status [37]. 

In contrast to our study, in Asian countries such as Taiwan, Thailand, and South Korea, low-income older adults depend more on younger members of their family for food-related decisions such as shopping and food preparation [38,39]. Our study, consistent with earlier studies in South Africa, showed that low-income older women, regardless of their food security status, were largely in control of food-related decisions in the household [40,41,42]. However, seemingly poor nutritional perceptions and preference for low-cost high energy-dense foods, compounded by financial constraints, seem to be associated with the low-quality diet. Almost two-thirds (62.8%) of the participants perceived that they consumed an adequate amount of healthy foods, and <20% indicated that they sometimes or often ate food not preferred (did not like). These findings showcase that our cohort can be influential in improving dietary quality for themselves and their households if they have the right nutritional perception and financial capacity to do so. However, the preference for low-cost unhealthy food might be a challenge.

In contrast to other studies in low-income communities in Africa in which food-insecure households were more likely to rely on informal food outlets, probably because food could be purchased on credit [13,43], all participants in the current study shopped in supermarkets due to their affordability [10]. Most women in the current study had access to a refrigerator in their homes (98.2%), which enabled them to preserve perishable products and this may explain the discrepancy in results between studies. Furthermore, women in the current study shopped at informal outlets, largely because of convenience (proximity to homes) of the spaza shops, and the availability of quality fresh fruits and vegetables at the street vendors, and not because of access to food on credit, which was less than 5% (data not shown.) Notably, as coping strategies to acquire food for the household, food-insecure participants indicated that they borrowed money for food mostly from friends, neighbours, and other places such as clubs and stokvels (informal savings or investment club). Increased social capital had been associated with reduced vulnerability to food insecurity in southern and eastern Africa [44,45]. Therefore, social capital (social networks, or groupings of people, which allow individuals to achieve things they could not on their own) in the form of friends, neighbours, and social clubs is an important safety net for accessing food for food-insecure groups. 

House ownership is prevalent in low-income urban areas in South Africa because the government, over the years, has made brick houses, most of which have basic amenities, available to low-income earners to alleviate poverty [46]. This should free up household income to be used for other essentials such as food. In accordance with previous findings [41], our results show that the majority (>50%) of household income is used for food regardless of food security status. Notwithstanding, our cohort, similar to previous studies, did not consume the recommended number of daily servings of vegetables, fruits, and dairy products regardless of their food security status and perception that they ate a healthy diet. This is understandable if a median household size of approximately five people is being fed with ZAR 1350/USD 84 monthly. This amounts to less than ZAR 10/USD 0.63 available for food per person per day. This apparent financial constraint possibly contributes to most (71%) worrying about their households having enough to eat (Supplementary Materials, Table S3) and opting for low-cost, high energy-dense foods. 

Consistent with earlier studies in South Africa, almost three-quarters (73%) of older women cared for at least one grandchild on a daily basis and financially supported at least three adults with the household income [47,48]. Older adults (≥60 years) in South Africa are eligible for a government social pension (old-age pension grant) of up to ZAR 1860/USD 101 to address poverty in older people [49]. This grant was the major source (76%) of household income for our cohort, the majority of whom were not married, suggesting that they were the main financial providers in their households of a median of five people, mostly adults [41]. The high rate of divorce, unemployment, HIV/AIDS-related death, out-of-marriage child-bearing, and teenage pregnancy in South Africa often result in the grandmothers stepping in to play an additional role as caregivers to their grandchildren in addition to supporting their adult children [50]. Although we did not show any differences between the food-secure and -insecure groups, earlier studies in South Africa have shown that being a recipient of a pension, financially supporting household members, especially children, and household size (number of people in the household), were all positively associated with food insecurity status of the grandmothers [10,32]. The current study shows that food-insecure women were from a higher housing density and used coping strategies (borrowing money more frequently, and eating less so that others, especially children in the household had enough food to eat) when compared to the food secure women. Additionally, we found that food-insecure women attended social/faith-based clubs, which may reflect a need for social, emotional, and financial support and to the provision of served meals [51,52]. High household density (overcrowding) in low-income communities has been linked to seeking social support, depression, poor well-being, and poor diet [53,54]. These results highlight the importance and need for social/faith groups in these low-income communities and these groups may be an appropriate target for food-insecure cohorts. 

### Strength and Limitations of the Study

This is the first study in South African to describe food security, dietary intake, and foodways in urban low-income older South African women. However, the small sample was purposively selected from low-income areas in Cape Town, and therefore, the results from this study cannot be generalised to other older adult women living in South Africa. Additionally, the food acquisition questionnaire did not include information on the quantity or amount spent on different food types purchased in the food outlets. Lastly, we only categorised the food security status dichotomously due to the small sample size, and this reduced sensitivity to detect differences in the levels of food insecurity. 

## 5. Conclusions

In our study, women who were food insecure consumed a greater percentage of their energy intake in the form of carbohydrates and less in the form of fats and presented with coping strategies to provide food for the household, which included borrowing money for food and eating less. Although the participants had access to basic amenities and refrigeration to preserve perishable food items, access to sufficient and quality food is a challenge. The low-quality diet of our cohort was associated with poor choices due to poor nutritional perceptions of sufficient consumption of fruits, vegetables, and dairy products despite actual low consumptions coupled with financial constraints, leading to poor food choices. However, as most of the women are responsible for food-related decisions, and the main breadwinner in their households, they can be instrumental in determining diet quality for themselves and their household. Sustainable avenues for alleviating the burden of financial care and promoting healthy food awareness and healthy living on budget through education campaigns might be three key options to improving the dietary choices, food security status, and overall well-being of low-income South African older women, which should be further explored.

## Figures and Tables

**Table 1:** Participant and household characteristics.

Variables	Total	Food Secure	Food Insecure	*p* Value
	*n* = 122	*n* = 77	*n* = 45	
Age (years)	67 (64–71)	66 (63–71)	68 (65–72)	0.109
Marital Status				
*Single/divorced/widowed*	113 (92.6)	72 (93.5)	41 (91.1)	
*Married/Living with partner*	9 (7.4)	5 (6.5)	4 (8.9)	0.625
Level of Education				
*No formal education/less than Grade 12*	113 (93.4)	70 (90.9)	43 (97.7)	
*Grade 12/tertiary*	8 (6.6)	7 (9.1)	1 (2.3)	0.256
*BMI (kg/m^2^)*	33.3 (29.2–40.5)	33.6 (29.2–42.8)	32.7 (28.9–36.7)	0.212
BMI Category				
*Normal weight (18.5–24.9 kg/m^2^)*	11 (9.0)	7 (9.1)	4 (8.9)	
*Overweight (25–29.9 kg/m^2^)*	24 (19.7)	10 (18.2)	14 (22.2)	0.863
*Obese (≥30 kg/m^2^)*	87 (71.3)	56 (72.7)	31(68.9)	
*Own their house*	100 (82.0)	61 (79.2)	39 (86.7)	0.302
Monthly household income				
*Less than ZAR 2500/USD 156*	92 (75.4)	56 (72.7)	36 (80.0)	
*More than ZAR 2500/USD 156*	30 (24.6)	21 (27.3)	9 (20.0)	0.368
Residential house type				
*Shack (informal house)*	13 (10.7)	7 (9.1)	6 (13.3)	
*Brick house*	109 (89.3)	70 (90.9)	39 (86.7)	0.464
*Housing density*	1.0 (0.6–1.5)	1.0 (0.5–1.3)	1.2 (0.8–1.7)	0.034 *
Household assets				
*Fridge*	120 (98.4)	75 (97.4)	45 (100)	0.276
*Microwave*	101 (82.8)	68 (88.3)	33 (73.3)	0.034 *
*Stove*	92 (75.4)	61 (79.2)	31 (68.9)	0.201
Basic amenities				
*Flush toilet system*	120 (98.4)	76 (98.7)	44 (97.8)	1.000
*Running water in house*	115 (94.3)	75 (97.4)	40 (88.9)	0.099
*Have access to electricity*	121 (99.2)	76 (98.7)	45 (100.0)	1.000
*Car ownership*	15 (13.5)	12 (16.9)	3 (7.5)	0.164

All data were reported as either *n* (%) or median (IQR–25–75th percentile). Chi-squared, Fisher’s exact, and Mann–Whitney U tests significant at * *p* < 0.05 were used to determine differences in categorical and continuous variables categorised by food security status, respectively. Abbreviation: BMI: Body mass index.

**Table 2:** Household and food decision characteristics of participants.

Variables	Total *n* = 122	Food Secure *n* = 77	Food Insecure *n* = 45	*p* Value
Number of people in the household	5 (3–6)	4 (2–6)	5 (3–7)	0.069
No. of adults supported by household income #	3 (2–4)	3 (2–4)	3 (2–4)	0.061
No. of children supported by household income	1 (0–3)	1 (0–2)	1 (1–3)	0.326
Care for their grandchildren on a daily basis	89 (73.0)	55 (71.4)	34 (75.6)	0.621
Monthly food expenditure (ZAR)	1350 (965–1900)	1375 (900–1915)	1310 (1000–1750)	0.917
Major source of household income				
*Pension/grant*	89 (73.0)	53 (68.8)	36 (80.0)	
*Others (Friends, family and business income)*	33 (27.0)	24 (31.2)	9 (20.0)	0.180
Participant decides what food to purchase	95 (77.9)	60 (77.9)	35 (77.8)	0.985
Participant is the main household shopper	64 (52.5)	39 (50.6)	25 (55.6)	0.601
Participant is responsible for food preparation	72 (59.0)	44 (57.1)	28 (62.2)	0.582
Participant is responsible for food budget	97 (79.5)	61(79.2)	36 (80.0)	0.918
Participants borrows money for food	55 (45.5)	29 (38.2)	26 (57.8)	0.036 *
Who participants borrow money for food from				
*Friends/neighbours*	55.6	51.7	60.0	
*Family*	9.3	6.9	12.0	
*Shop owners*	3.7	3.4	4.0	
*Others (stokvels and other clubs)*	31.5	37.9	24.0	0.830
Participant eats less so children in the household have more to eat	50 (41.0)	21 (27.3)	29 (64.4)	0.001 *
Shop at supermarkets	122 (100)	77 (100.0)	45 (100.0)	
Shop at spaza shops	107 (88.4)	66 (86.8)	41 (91.1)	0.478
Shop at street vendors	90 (73.6)	57 (73.7)	33 (73.3)	0.839
Shop at fast food outlets	66 (54.1)	46 (59.7)	20 (44.4)	0.102
Go to social/faith-based clubs	85 (70.2)	48 (63.2)	37 (82.2)	0.027 *

All data were reported as either *n* (%) or Median (IQR–25–75th percentile). Chi-squared, Fisher’s exact, and Mann–Whitney U tests significant at * *p* < 0.05 were used to determine differences in categorical and continuous variables categorised by food security status, respectively. # The number of adults and people in the household includes the participants.

**Table 3:** Neighbourhood food environment and food acquisition characteristics of low-income older South African women.

Variables	Supermarkets	Spaza	Street Vendor	Fast Foods	Social/Faith-Based Clubs
Spending per month (Rand)	1000 (700–1500)	160 (60–400)	140 (50–240)	105 (50–200)	10 (0.0–50)
Spending per month (USD)	66.7 (46.7–100)	10.7 (4–26.7)	9.3 (3.3–20)	7 (3.3–13.3)	0.7 (0–3.3)
Frequency of visits					
*>once a week*	12 (9.9)	77 (63.6)	30 (24.8)	2 (1.7)	53 (43.8)
*2–4 times a month*	60 (49.6)	27 (22.3)	39 (32.2)	14 (11.6)	24 (19.8)
*Once a month*	50 (40.5)	3 (2.5)	20 (16.5)	50 (41.3)	8 (6.6)
*Never*	0	14 (11.6)	33 (26.4)	55 (45.5)	36 (29.8)
Frequently purchased food items#	Dry grains/cereals & legumes 117 (95.9)	Bread 95 (87.9)	Fresh/frozen veg 69 (77.8)	Chicken and chips 38 (57.6)	Bread/sandwiches 46 (54.0)
	Frozen meat/chicken 112 (91.8)	Dairy 52 (47.7)	Fresh or frozen fruits 61 (68.9)	Fish and chips 32 (48.5)	Vegetable and salad 59 (68.2)
	Fresh and frozen veg 107 (87.7)	Condiments 43 (40.0)	Meat 16 (17.8)	Burger and chips 7 (10.6)	Meat 55 (63.5)
	Dairy 103 (84.4)	SSBs 29 (27.1)			Potato/pasta or rice 54 (62.4)
	Fat and oil 103 (84.4)				
	Eggs 99 (81.0)				
	Fish 80 (65.6)				
	SSBs 80 (65.6)				
	Snacks 76 (62.3)				
	Bread 75 (61.5)				
	Fresh and frozen fruits 74 (60.7)				
Major Reason for shopping at food outlets (%) #	Price (61.2)	Convenience (88.5)	Quality (32.2)	Variety (96.9)	Socialise & fellowship (97.6)
Transportation mode #					
*Walk*	47 (38.0)	108 (100.0)	82 (92.2)	33 (50.0)	57 (63.5)
*Public transport*	63 (52.2)	0	5 (5.6)	27 (40.9)	32 (35.3)
*Private car*	12 (9.9)	0	2 (2.2)	6 (9.1)	1 (1.2)

Data reported as median (IQR–25–75th percentile) or *n* (%). # Expressed as median/percentage of those participants who buy from the outlets. Abbreviation: Veg: Vegetables; SSBs: Sugar-sweetened beverage.

**Table 4:** Energy distribution of macronutrients and food intake of 12 selected food groups for urban older low-income South African women.

	AMDR	Food Secure (*n* = 69)		Food Insecure (*n* = 40)		
		Median	P25–P75	Median	P25–P75	*p*-Value
**Macronutrients**						
Energy intake (kcal)	1600	1883	1461.4–2367.8	1793.1	1387.1–2359.0	0.596
%TE from protein	10–35	12.4	11.0–13.8	12.6	10.3–14.0	0.886
%TE from total fat	20–35	23.5	18.7–26.2	19.0	16.3–22.0	0.003 *
%TE from total carbohydrate	45–65	63.2	59.6–68.5	67.8	63.9–70.3	0.013 *
**Food intakes**						
%TE from fruits		7.1	3.2–11.2	5.7	2.6–10.4	0.314
%TE from vegetables		2.4	1.5–4.7	2.8	1.6–6.1	0.514
%TE from cooked porridge		7.8	4.3–15.2	9.8	6.1–17.8	0.199
%TE from starchy grains		29.2	22.2–38.1	30.9	24.1–40.0	0.593
%TE from legumes		1.4	0–2.7	2.1	1.1–4.0	0.049 *
%TE from nuts and seeds		0	0–2.0	0	0–2.0	0.719
%TE from milk and dairy products		7.9	3.8–13.5	7.1	3.2–9.8	0.423
%TE from animal protein foods		10.4	6.7–13.6	8.1	4.8–12.8	0.068
%TE from sugar and sugary foods		14.8	9.2–19.6	15.5	10.9–22.3	0.134
%TE from fats and oils		4.3	1.3–8.6	2.3	1.1–5.3	0.023 *
%TE from savoury snacks, dishes and sauces		0.9	0–2.0	0.7	0-2.1	0.392
%TE from alcohol		0	0	0	0	0.553

Abbreviations: %TE: percentage of total energy; %: percentage; AMDR = acceptable macronutrient distribution range); P25-P75: 25th percentile–75th percentile. * *p*-Values determined through Mann–Whitney U test.

**Table 5:** Consumption of selected food groups by urban older low-income South African women.

			Food Secure (*n* = 69)		Food Insecure (*n* = 40)		
Variables	Total Consumed *n* (%)	* Meet Consumption Recommendation	No of Servings Per Day Median	P25–P75	No of Servings Median	P25–P75	*p*-Value
Fruits	109 (99.1)	28 (26.2)	1.4	0.5–2.7	0.8	0.3–1.5	0.166
Vegetables	109 (99.1)	15 (13.9)	1.2	0.8–2.0	1.6	0.7–2.9	0.555
Milk and dairy products	103 (93.6)	6 (5.5)	1.3	0.6–2.0	1.2	0.6–1.5	0.549
Cooked porridge	107 (97.3)		1.5	0.7–2.6	1.6	0.9–2.3	0.328
Starchy grains	110 (100)		5.3	3.8–8.0	5.2	4.0–7.2	0.714
Legumes	80 (73.4)		0.3	0.0–0.6	0.4	0.2–0.8	0.120
Animal protein foods	110 (100)		1.1	0.8–1.8	0.8	0.5–1.5	0.188
Nuts and seeds	43 (39.1)						

Abbreviations: TE: total energy; %: percentage; P25-P75: 25th percentile–75th percentile. *p*-Values determined through Mann–Whitney U test. * Recommended number of servings (at least two servings of fruits, three servings of vegetables, and three servings of dairy products) https://www.nia.nih.gov/health/serving-and-portion-sizes-how-much-should-i-eat (accessed on 24 July 2020).

## Data Availability

The supporting data for this study are available from the corresponding author upon reasonable request.

## Supplementary Materials

The following are available online at https://www.mdpi.com/article/10.3390/ijerph18083973/s1, Table S1: Food acquisition questionnaire for older South African adults, Table S2: The content of the 12 food groups, Table S3: Percentage distribution of participants’ responses to the HFIAS questions, Table S4: Perceptions of healthy food consumption of low-income older South African women.
