# Supplementary material for: Food Security, Dietary Intake, and Foodways of Urban Low-Income Older South African Women: An Exploratory Study
Source: Int J Environ Res Public Health. 2021 Apr 9;18(8):3973. doi: 10.3390/ijerph18083973 (PMC8069086; doi:10.3390/ijerph18083973)
Supplement: Supplementary file 1 [file ijerph-18-03973-s001.pdf]

Table S1: Food Acquisition Questionnaire For Older South African Adults

Item	Response/s
About you and your family	
1) How many people (including you) are living in your household?	______ Number of children (<18 yrs.)<br>_______Number of adults
2a) How many rooms (bedrooms and living room) do you have in your house?<br>2b) Does your family own or rent your home?	_______Number of rooms<br>______Own/rented
3) Which of the following best describes the home in which you live?(tick only one)	_____ Single room<br>_____ Informal dwelling or shack<br>____ Backyard room or flat<br>_____Flat or semi-detached house<br>_____Free-standing house (brick or concrete)<br>____ Other
4) Main source/s of household income (tick only one)	_____ Salary/salaries of family members<br>_____ Own business income<br>_____ Pension/Grant<br>_____ Family and friends<br>_____ Donations<br>_____ Other: ______________________________
5) Other sources of household income (tick all that apply):	______ Salary/salaries of family members<br>_____ Own business income<br>______ Pension/Grant<br>_____ Family and friends<br>_____ Donations<br>____ _ Other:_____________________________
Household Shopping and Foodways	
6) In your household, who mostly decides what type of food to buy? (tick only one)	____ Myself<br>____ My partner/Spouse<br>__ _ My children<br>____ Other members of my family/household<br>___ _ Other: ______________________________
7) In your household, who mostly decides how much money is spent on food?(tick only one)	____ Myself<br>____ My partner/Spouse

	____ My children<br>____ Other members of my family/household<br>____ Other: _______________________________
8) In your household, who is mostly responsible for preparing the food?(tick only one)	____ Myself<br>____ My partner/Spouse<br>____ My children<br>____ Other members of my family/household<br>____ Other: _______________________________
9) In your household, where do you store your perishable food? (Place 1, 2, 3 next to the answers which best describe where you store your food, 1 is the MOST important reason).)	_____Fridge<br>____ Freezer<br>____ Cupboards<br>____ Buckets<br>____ Tupperware/plastic containers with lids<br>____ Vegetable rack<br>____ Coolbox<br>____ At a neighbour/friend/family member<br>____ Other: _____________________________
10) On average, how much money is spent on food in a month in your household?	R_____________/month<br>_______ I don't know
11) In your household, who mostly does the shopping for food? (tick only one)	____ Myself<br>____ My partner/Spouse<br>____ My children<br>___ Other members of my family/household<br>____ Other: _______________________________

	**Supermarket**	**Spaza/Convenience Store**	**Vendor/ Tshisanyama**
i) **How many times** per week or month do you buy food at this type of venue?Q_12	_____ times per week or<br>_____ times per month	_____ times per week or<br>_____ times per month	_____ times per week or<br>_____ times per month
ii) How do you **get to and from** this venue?	___ Walk<br>____Bus<br>___ Train<br>___ Taxi<br>___ Private car<br>___ Walk + Transport	___ Walk<br>___ Bus<br>____Train<br>___ Taxi<br>___ Private car<br>___ Walk + Transport	____ Walk<br>____ Bus<br>____ Train<br>____ Taxi<br>___ Private car<br>____ Walk + Transport
iii) Which **types of foods** do you buy at this venue? (Tick all that apply)	_____ Fresh /frozen fruits<br>____ Canned fruits<br>____ Fresh/ frozen vegs	__ Fresh /frozen fruits<br>___ Canned fruits<br>____ Fresh/ frozen vegs	_____ Fresh /frozen fruits<br>_____ Canned fruits<br>_____ Fresh/ frozen vegs

	____ Tinned vegetables<br>____ Bread<br>____ Maize/mielie meal<br>____ Meat/chicken<br>____ Processed meat<br>____ Fish/Tinned fish<br>____ Dairy and cheeses<br>____ Fats/oils/margarine<br>_____Eggs<br>_____ Beans/samp<br>_____Snacks and sweets<br>_____ Cool drinks<br>_____Fruit juices<br>_____ Alcohol	_____ Tinned vegetables<br>_____ Bread<br>_____ Maize/mielie meal<br>_____ Meat/chicken<br>_____ Processed meat<br>_____ Fish/Tinned fish<br>_____ Dairy and cheeses<br>_____ Fats/oils/margarine<br>_____ Eggs<br>_____ Beans/samp<br>_____ Snacks and sweets<br>_____ Cool drinks<br>_____ Fruit juices<br>_____ Alcohol	_____ Tinned vegetables<br>_____ Bread<br>_____ Maize/mielie meal<br>_____ Meat/chicken<br>_____ Processed meat<br>_____ Fish/Tinned fish<br>_____ Dairy and cheeses<br>_____ Fats/oils/margarine<br>_____ Eggs<br>_____ Beans/samp<br>_____ Snacks and sweets<br>_____ Cool drinks<br>_____ Fruit juices<br>_____ Alcohol
iv) How **much money do you spend on average** each time you shop at this venue?	R ____________<br>____ Don't know	R ____________<br>_____ Don't know	R ____________<br>______ Don't know
v) **Why do you choose** to shop at this venue? (choose only one)	_____ Price/Value for money<br>_____ Close to where I stay<br>_____ Quality<br>_____ Cleanliness<br>_____ Variety<br>_____ I am well treated<br>_____ I am given credit<br>_____ Other: _____________	_____ Price/Value for money<br>_____ Close to where I stay<br>_____ Quality<br>_____ Cleanliness<br>_____ Variety<br>_____ I am well treated<br>_____ I am given credit<br>_____ Other: _____________	_____ Price/Value for money<br>_____ Close to where I stay<br>_____ Quality<br>_____ Cleanliness<br>_____ Variety<br>_____ I am well treated<br>_____ I am given credit<br>_____ Other: _____________

	**Fast Food**	**Restaurant**	**Club/Church meeting**	**Relative or friend**
i) How many times per week do you eat a meal at this type of venue?	_____ times per week or<br>_____ times per month	_____ times per week or<br>_____ times per month	_____ times per week or<br>_____ times per month	_____ times per week or<br>_____ times per month
ii) How do you get to and from this venue?	____Walk<br>____ Bus<br>____ Train<br>____ Taxi<br>____ Private car<br>____ Walk + Transport	_____ Walk<br>_____ Bus<br>_____ Train<br>_____ Taxi<br>_____ Private car<br>_____ Walk + Transport	_____ Walk<br>_____ Bus<br>_____ Train<br>_____ Taxi<br>_____ Private car<br>_____ Walk + Transport	_____ Walk<br>_____ Bus<br>_____ Train<br>_____ Taxi<br>_____ Private car<br>_____ Walk + Transport
iii) Which types of foods do you eat a meal at this venue?	_____ Fried chicken & chips<br>_____ Burgers & chips<br>____ Gatsbys/Sandwiches<br>_____ Fish & chips<br>_____ Curries/Stews/Bredies<br>____ Other:____________	____ Meat/fish/chicken<br>____ Vegetables/Salads<br>____ Potatoes/Rice/Pastas<br>____ Maize/mielie meal<br>____ Dairy/Cheese<br>____ Curries/Stews/Bredies	_____ Meat/fish/chicken<br>_____ Vegetables/Salads<br>____ Potatoes/Rice/Pastas<br>____ Maize/mielie meal<br>____ Dairy/Cheese<br>____ Curries/Stews/Bredies	____ Meat/fish/chicken<br>____ Vegetables/Salads<br>____ Potatoes/Rice/Pastas<br>____ Maize/mielie meal<br>____ Dairy/Cheese<br>____ Curries/Stews/Bredies

	_____ Other:____________	_____ Other: _____________<br>_____ Other: _____________	_____ Other: _____________<br>_____ Other: _____________	_____ Other: _____________<br>_____ Other: _____________
iv) How much money do you spend on average when you eat a meal at this venue?	R ____________<br>______ Don't know	R ____________<br>_____ Don't know	R ____________<br>______ Don't know	R ____________<br>_____ Don't know
v) Why do you choose to eat a meal at this venue? (Choose only one)	__1___ Price/Value for money<br>__2__ Close to where I stay<br>__3__ Quality<br>__4__ Cleanliness<br>__5__ As a treat<br>__6___ I am well treated<br>__7__ I am given credit<br>__8__ Socialise/Fellowship<br>__9__ Other: _____________	_____ Price/Value for money<br>_____ Close to where I stay<br>_____ Quality<br>_____ Cleanliness<br>_____ Variety<br>_____ I am well treated<br>_____ I am given credit<br>_____ Socialise/Fellowship<br>_____ Other: _____________	_____ Price/Value for money<br>_____ Close to where I stay<br>_____ Quality<br>_____ Cleanliness<br>_____ Variety<br>_____ I am well treated<br>_____ I am given credit<br>_____ Socialise/Fellowship<br>_____ Other: _____________	_____ Price/Value for money<br>_____ Close to where I stay<br>_____ Quality<br>_____ Cleanliness<br>_____ Variety<br>_____ I am well treated<br>_____ I am given credit<br>_____ Socialise/Fellowship<br>_____ Other: _____________

**Coping strategies**	
13) How **often do you borrow** food or money for food in a month?(tick only one)	_____ Almost never/never<br>_____ One time<br>_____ 2-3 times<br>_____ Almost every week<br>_____ More than once per week
14) if you **borrow money for food**, from **whom** do you borrow? (tick all that apply)	_______Friends and/or neighbours<br>______Family/children<br>______Shop owners<br>_______Others: ____________________________
15) Do you eat **less than you would like**, so there will be enough for other members of your households, especially the children?)	____Often true<br>____Sometimes true<br>____Never true<br>_____Don't know/Refused

**Do you think you have a healthy diet?**	
18 Think about all the food you eat on a regular basis, would you say you **have a healthy diet**?	______ Yes ____No____ Don't Know
19) If you compare your diet to a "healthy diet", do you eat enough fruit?	___ Too little ____About right _____ Too much
20) If you compare your diet to a "healthy diet", do you eat enough vegetables?	__ Too little _____About right _____ Too much
21) If you compare your diet to a "healthy diet", do you eat enough meat/fish/chicken?	____ Too little ___About right _____ Too much
22) If you compare your diet to a "healthy diet", do you eat enough dairy products?	___ Too little ____About right _____ Too much
23) If you compare your diet to a "healthy diet", do you eat enough breads/grains/cereals?	____ Too little ____About right ____ Too much

**Definition of shopping areas/venues in South Africa**	
Supermarkets 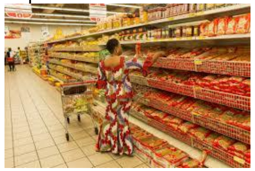	Recognized as retail store chains such as Pick n Pay, Spar, Checkers, Shoprite in South Africa that offer a broad selection of foods and household products.
Convenience shops/Spaza 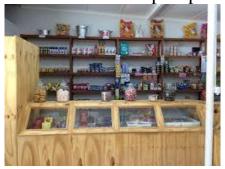	Small retail stores, often in a residential area, that carry a limited selection of items such as staples, junk food, and drugstore items, and which is open long hours for the convenience of shoppers. Spazas are micro-convenience stores that mostly operate in low-SES communities.
Street vendors 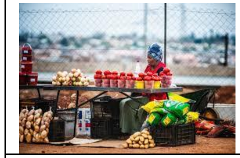	Persons who offer goods or services for sale to the public without having a permanently built structure but with a temporary static structure or mobile stall (or head-load).
Fast food outlets 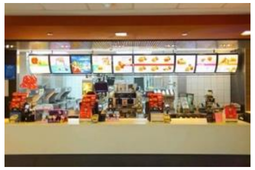	These outlets offer foods or meals that are prepared or ready for immediate consumption either at the place of purchase or elsewhere. They are also known as takeaway outlets.

Food Category	Definition
Fresh /frozen fruits	Any fruit based item: fresh, frozen, dried, whole, cut-up or pureed
Canned fruits	Any fruit based item: canned
Fresh/ frozen vegetables	Any fresh or frozen vegetable product such as carrot, spinach, tomatoes, onions, broccoli, potatoes, butternut, beetroot, peas and corn
Tinned vegetables	Any tinned vegetable product (not including beans)
Bread	All types of breads
Cereal and cereal products	All whole and refined grains such as maize, wheat (flour), rice, oats, cornmeal, barley or another cereal grain, pasta, oatmeal, breakfast cereals, porridges and tortillas, wraps, potato, two minute noodles.
Beans	Dried beans, split peas, lentils, chickpeas broad beans, soya beans, kidney beans, canned kidney beans, haricot, lentils, chickpeas, lima beans, soybeans, and others
Snacks and sweets	High-energy baked goods, ready-to make/Ingredients, frozen treats, milk-based desserts, candy and sweet toppings with a sweet or salty taste. These include chocolates, chips, biscuits, donuts, cookies and others. Nuts, peanuts, Energy bars, crackers , muffins, sugar (all types), honey, syrup, ice cream, trail mixes, droewors ice-cream
Processed meats and deli	Cold cuts, viennas, tinned meats, meat pies, chicken nuggets, samosas, breaded fish fingers, bacon, canned meat,, tinned chicken, smoked chicken etc.
Dairy products	All type of food produced from containing the milk such as yogurt, cheese, and butter whether low or high in fats, fresh, frozen, canned or processed, and dairy alternatives e.g. Soy milk –if fortified
Meat and meat products	All meat types: red meat (beef, lamb, goat, poultry (chicken and turkey) and pork whether fresh, frozen, dried, whole, cut-up - incl boerewors, tripe, liver excluding canned meat
Fish and seafood	All fish types and other seafood such as shrimps whether, fresh, frozen, dried, whole, cut-up
Canned fish	All tinned fish e.g. pilchards, tuna in tomato sauce etc.
Egg	All types of eggs : farm fresh, free range etc.
Fruit juices (100%)	All fruit juices (100% fruit juices, tetra pack types, etc.)
Alcohol	Any fermented liquor, such as wine, beer, or distilled spirit, that contains an intoxicating agent.
Fats and oils and margarine	All types of margarine (brick and tub), cooking oils, mayonnaise, and salad dressings, creams etc. including peanut butter
Fast foods and street food	Fast foods are usually prepared meals that are purchased from fast food outlets such as KFC, while street foods are purchased from vendors

Table S2: The content of the twelve food groups

	Food group	Definition
**1**	Fruits	All fresh fruit, dried fruit, unsweetened fruit juice
**2**	Vegetables	All vegetables
**3**	Cooked porridge	Cooked porridge
**4**	Starchy grains	Breakfast cereals, potato, sweet potato, rice, pasta, bread, vetkoek, etc
**5**	Legumes	Legumes, soup made with lentils
**6**	Nuts and seeds	Nuts, peanuts, peanut butter
**7**	Milk and Dairy products	All milk and milk products such as sour milk (maas), cheese and yoghurt
**8**	Animal protein foods	meat, chicken, fish, eggs
**9**	Fats and oils	Oils, margarine, salad dressings, etc
**10**	Sugar and sugary foods	Sugar, sweets, chocolate, cake, biscuits, pudding, cold drinks, sweetened fruit juice, canned fruit.
**11**	Savoury snacks, dishes and sauces	Salty snacks (crisps, nik-naks, etc), meat pies, samosas, pizza, tomato sauce, chutney
**12**	Alcohol	All home-made and commercial alcoholic drinks

Adapted from Wentzel-Viljoen et al, 2018

Table S3: Percentage distribution of participants' responses to the HFIAS questions

			Frequency		
	**HFIAS Questions**	**Yes**	**Rarely**	**Sometimes**	**Often**
	Did you or your household members due to lack of resources in the last 30 days:			%	
Q1	Worry about food shortage in their household?	71.7	16.7	20.8	34.2
Q2	Not able to eat foods they preferred?	45.5	9.1	15.7	20.7
Q3	Ate just a limited variety of foods?	59.3	19.5	24.6	15.3
Q4	Ate foods that are not preferred?	42.1	13.2	14.9	14.0
Q5	Ate smaller meal than required due to insufficient amount of food	51.2	15.7	20.7	14.9
Q6	Ate fewer meals in a day	46.7	10.7	23.0	13.1
Q7	Had no food at all in the house	32.0	10.7	9.0	12.3
Q8	Went to bed hungry because there was not enough food	23.0	10.7	6.6	5.7
Q9	Went a whole day & night without eating.	9.0	4.1	3.3	1.6

Data is presented as proportions (%) ; Abbreviations: HFIAS: Household food insecurity access scale; Q: Question

Table S4: Perceptions of healthy food consumption of low-income older South African women

Variables	Overall N=122	Food secure N=77	Food insecure N=45	P value
Would you say you have a healthy diet?				
Yes	76 (62.8)	50 (65.8)	26 (57.8)	
No	24 (19.8)	13 (17.1)	11 (24.4)	
Don't know	21 (17.4)	13 (17.1)	8 (17.8)	0.587
Compare your diet to a "healthy diet", do you eat enough fruits?				
Too little	39 (32.2)	19 (25.0)	20 (44.4)	
About right	41 (33.9)	28 (36.8)	13 (28.9)	
Too much	41 (33.9)	29 (38.2)	12 (26.7)	0.084
Compare your diet to a "healthy diet', do you eat enough vegetables?				
Too little	16 (13.2)	7 (9.2)	9 (20.0)	
About right	40 (33.1)	26 (34.2)	14 (31.1)	
Too much	65 (53.7)	43 (56.6)	22 (48.9)	0.237
Compare your diet to a "healthy diet", do you eat enough dairy products?				
Too little	45 (37.5)	32 (42.1)	13 (29.5)	
About right	44 (36.7)	23 (30.3)	21 (47.7)	
Too much	31 (25.8)	21 (27.6)	10 (22.7)	0.153

Data are presented as proportions n(%). P-values determined through chi squared test
